# Risk of self-injurious behavior increases in adolescence: new findings

**DOI:** 10.3389/fpsyg.2025.1563027

**Published:** 2025-10-03

**Authors:** Angélica Quiroga-Garza, Fátima Ochoa Vera, Ana Lucía de la Garza Chapa, Paola Abigail Ibarra Almaguer, Elisa Becerra Pérez, Mariana Hernández Hernández, Ana Gabriela Durán Treviño

**Affiliations:** Universidad de Monterrey, Garza García, Mexico

**Keywords:** self-injury, childhood traumatic events, emotional dysregulation, suicidal ideation, peer influence, digital social network influence

## Abstract

**Introduction:**

Self-injurious behaviors are a type of self-inflicted violence, with or without suicidal intentions. Approximately 10% of the global population has exhibited non-suicidal self-injury (NSSI); in Mexico, these behaviors are most common among individuals aged 10–29, with an average onset age of 12.3 years in Nuevo León.

**Objective:**

To determine the predictive power of childhood traumatic events, emotional dysregulation, peer social pressure, and digital social networks on the incidence of self-injurious behaviors in adolescents aged 11–16 who are part of the educational system in the metropolitan area of Nuevo Leon, Mexico.

**Methods:**

A quantitative, non-experimental, cross-sectional, explanatory study was conducted. Six measurement questionnaires were used including a Screening of Self-injurious Behaviors and Suicide Related with Internet Use for adolescents designed by the authors for this study. For statistical analyses, we were considered ratio or continuous variables. The correlation was carried out using Spearman's rank coefficient and a significance level of 95% was applied to determine the predictive value of each variable on self-injurious behaviors and/or suicidal ideation.

**Results:**

A high prevalence of the self-injurious behaviors and suicide ideation among adolescents, highlighting not only traditional risk factors like emotional dysregulation, peer pressure, and childhood trauma but also the significant impact and predictive value of digital exposure to content about self-harm and suicide in social digital networks.

**Conclusion:**

Not all adolescents fully recognize the severity of their own behaviors or their peers' behaviors, which can lead to delays in seeking help. There is a need for prevention efforts, such as psychoeducation and open communication, alongside regulation of harmful content on social media. To address the problem holistically, collaboration between parents, educators, and health professionals is recommended.

## Introduction

The increase in self-injurious behaviors among adolescents has become a significant issue affecting not only the individual engaging in these behaviors but also their families, peer groups, and communities (Pan American Health Organization, 2024). Halicka and Kiejna (2018) reported that approximately 10% of the global population has experienced non-suicidal self-injury (NSSI) at some point in their lives. By 2022, 17% of the global population was estimated to engage in self-injury at some point in their lifetime (Hull, 2020). Among adolescents, between 13% and 45% have exhibited self-injurious behaviors, with these being more common in females. Among young adults, the prevalence ranges from 5% to 35% (Campo Arias, 2022; Plener et al., 2016; Vega et al., 2018). This issue has gained attention as a public health priority, with international organizations collaborating to prevent youth suicide. For instance, the World Health Organization (WHO) (2021), in its Comprehensive Mental Health Action Plan 2013-2020, established a global goal (No. 3.2) to reduce suicide rates by one-third by 2030.

Adolescents are multidimensional beings with various surrounding systems that act as forces motivating their decision-making. All of this, further highlights the importance of an integral approach, considering adolescents exhibiting self-injurious behaviors, their past experiences (*history of childhood trauma*), horizontal relationships (*social pressure*), use of technology (*social networks*), and their affectiveness (*emotional regulation*). Based on this, our research investigates the predictive relationship between different personal and social variables and self-injurious behaviors in adolescence, allowing for deeper analysis.

### Self-injury and associated factors

Self-injurious behaviors encompass any form of self-inflicted violence and can be categorized into two main groups: non-suicidal self-injury (NSSI) and self-destructive behaviors associated with suicidal thoughts, ideation, and attempts (SANE Australia, 2023; Villalobos-Fajardo, 2019). NSSI refers to the deliberate infliction of harm to the body's surface without suicidal intent (Fleta Zaragozano and Miral, 2017; Gold and Frierson, 2020; Healey, 2017; Lurigio et al., 2024). Its primary purpose is emotional regulation and the management of disruptive thoughts, often related to suicidal ideation, but without leading to lethal behavior (Plener et al., 2016; Vega et al., 2018; Villalta et al., 2018). Common methods include pinching, biting, cutting, hitting, scratching, hair-pulling, and burning (Obando et al., 2018; Pérez et al., 2021).

Throughout history, a variety of terms have been used to refer to NSSI: parasuicide, destructive behavior, self-mutilation, self-injurious, self-harm. Self-injurious is defined as any act of self-ham without explicit suicidal intent (Norman et al., 2020). The differentiation between suicidal ideation and NSSI is the presence or absence of intent to die; both are similar in that they are forms of deliberate self-harm. Main factors of differentiation are function, lethality, medical severity, prevalence rate, and frequency (Clarke et al., 2019; Nock, 2017). On one hand, NSSI are considered adaptive behaviors in which a person deliberately inflicts physical harm upon themselves as a mechanism for self-relief, without any desire to die, for different reasons including releasing tension, expressing themselves, feeling uniqueness, influencing others, and feeling control (Del Rosario et al., 2018). On the other hand, individuals with a single suicide attempt are more prone than individuals with suicidal ideation to suffer from a depressive or disorder, post-traumatic stress disorder, substance abuse disorder, or have suffered or presented some type of abuse, whether violent, psychological, or sexual. Furthermore, individuals with multiple suicide attempts show a high rate of family history of suicide and childhood emotional abuse, as well as psychopathological characteristics, a greater degree of suicidal ideation, and a higher proportion of psychiatric disorders (Park et al., 2020).

In line with previous research (Beckman et al., 2016; Obando et al., 2018; Runeson et al., 2016), a study with Mexican adolescents with and without suicidal ideation showed that 64.8% of those who engage in some type of self-harm have a significant risk of suicide. A 52.1% of adolescents with a suicide attempt reported having engaged in self-injurious behaviors. These findings indicate that both suicidal ideation and the diversity of self-injurious behaviors are significant predictors of suicide attempts, and both are associated with depression and anxiety.

Although NSSI does not inherently predict suicide, it is closely associated with suicidal ideation and even suicidal acts; therefore, the identification of self-injurious behaviors should be interpreted as a variable for early intervention (Duarte et al., 2020).

Self-injurious behaviors typically begin in mid-adolescence (De Luca et al., 2023; Steinhoff et al., 2020, 2021). Their long-term consequences can include increased psychological distress (Buelens et al., 2019). While NSSIs differ from suicide in lethality, methods, cognition, and intent, adolescents engaging in these behaviors are at a higher risk for suicidal thoughts and attempts than those who do not (Jacobson and Gould, 2007). Suicide is the second leading cause of death globally among individuals aged 10–24 (Kruzan and Whitlock, 2022) and the fourth leading cause of death in Mexico among those aged 15–29. In Mexico, self-injurious behaviors are most common among individuals aged 10–29 [Instituto Nacional de Estadística Geografía (INEGI, 2021; Instituto Nacional de Estadística y Geografía (INEGI), 2024].

Among the risks associated with self-injurious behaviors are physical and psychological symptoms. Among physical risks are headaches (Tsai et al., 2011), multiple sleep variables— short sleep duration, insomnia symptoms, poor sleep quality, sleep insufficiency, unrefreshed sleep, sleep dissatisfaction, daytime sleepiness, fatigue, snoring, and nightmares—(Liu et al., 2017), scarring (Burke et al., 2016), visible wounds of frequent cuts, burns, bruises, scratches, infections and other physical injuries (Fenkel, 2022). Some of the psychological symptoms are anxiety, depression, and stress levels, as well as susceptibility to suicidal ideation, attempts, and NSSI, particularly in children and adolescents, all of them increased after the pandemic-induced confinement and social distancing due to SARS-CoV-2 (Farooq et al., 2021; Mancebo, 2022).

Furthermore, emotional states are a product of the meaning given to events, that is, brain predictions that connect bodily states to events (Šimić et al., 2021). So, if strategies to manage emotions—with the aim of avoiding or experiencing unpleasant sensations—are inadequate or insufficient, the emotion intensifies, impeding its understanding and initiating a dysregulation vicious cycle that affects social and psychological functioning (Bonet et al., 2020; Vargas and Muñoz, 2013). Additionally, adolescents often resort to avoidance through NSSI in stressful, potentially traumatic situations (Gratz et al., 2018). This allows them to compensate for the unpleasant state in the short term with a temporary calm and relief (Klonsky, 2009), a desperate way to self-regulate leading to dependency and repeated occurrences (Esposito et al., 2023; Walsh, 2012), a risk to their physical and mental health (Bautista Hernández et al., 2022; Hervás and Moral, 2017; Peh et al., 2017; Wu et al., 2021).

Other contributing factors include parenting styles, attachment issues, childhood trauma, and family dynamics. Studies have linked insecure attachments and family violence to emotional dysregulation, low self-esteem, and self-aggression in adolescents (Romero-Acosta et al., 2021). Factors such as emotional distance, abuse, neglect, and low parental regulation—considered traumatic childhood events, ACEs—further increase vulnerability (Fischer et al., 2022; Romero-Acosta et al., 2021; Villalta et al., 2018), given that it is difficult for children and adolescents to help themselves or resolve what is happening, which leads them to lose trust in those around them and in themselves (Corral-Proaño and Díaz Mosquera, 2019; Ford and Courtois, 2009). ACEs have both a physical and cognitive effect (Del Castillo Drago et al., 2021), and are associated with a variety of risk behaviors such as criminal acts, drug use, alcohol abuse, self-harming behaviors, risky sexual behaviors, and internet addiction (Franco et al., 2020; Ménard and MacIntosh, 2021). The aim is to obtain gratifying sensations despite their negative consequences (Babad et al., 2021; Burgos-Ocasio and Pinilla-Díaz, 2019; Cao et al., 2021; Franco et al., 2020; Scheidell et al., 2018). For each ACE presented, a 42% increase in any risk behavior has been observed (Garrido et al., 2018).

### Peer and social media pressure

During adolescence, a period of significant social, emotional, and cognitive change, peer acceptance plays a critical role. Rejection can lead to risky behaviors, and peer pressure may act as a social contagion, with adolescents mimicking observed behaviors (Brieant et al., 2018).

Exposure to self-injurious behaviors by close friends or family increases the risk of imitation (Copeland et al., 2019; Hasking et al., 2013; Jarvi et al., 2013; Smith-Gowling et al., 2018). This pressure can turn into social contagion as individuals replicate what they observe in their environment.

Today, this “social contagion” has reached new dimensions, spreading increasingly within the virtual world. Social media amplifies this contagion by providing spaces where adolescents encounter content that normalizes or glorifies self-injury (Lavis and Winter, 2020). Adolescents who tend to self-harm reported that images of others' self-injuring on the internet were the main reason they initiated these behaviors (Dekel et al., 2024; Jacob et al., 2017; Lavis and Winter, 2020).

Therefore, peers pressure on self-injurious behavior among adolescents has been little studied, and further research is needed (Bentley et al., 2014; Smith-Gowling et al., 2018).

Social networks have created a new space where adolescents are constantly exposed to different types of topics and content. The possibility of sharing and creating communities with similar thoughts fosters a sense of group belonging and encourages adolescents through positive reinforcement (Lavis and Winter, 2020; Xeni, 2019; Yearwood et al., 2021). When other young people with NSSI interact with shared posts, they mutually validate each other. This has been found on social networks such as Tumblr, Twitter, Reddit, and Instagram (Brown et al., 2020; Guccini and McKinley, 2022; Houston, 2017; Lavis and Winter, 2020). Challenges like the “Blue Whale” and communities sharing such content exacerbate the problem (Gámez-Guadix et al., 2022).

Increased accessibility to harmful online content contributes to desensitization, making the internet a significant influence on self-injurious behaviors and suicidal ideation (Biernesser et al., 2020).

### Present study

This research seeks to find the related values of variables on self-injurious behaviors in adolescence. Adolescents are multidimensional beings with various surrounding systems that act as forces motivating their decision-making, which further highlights the importance of an integral approach, considering their past experiences (history of childhood trauma), horizontal relationships (social pressure), technology (social networks), and their emotional responses (emotional regulation). We hypothesize that emotional dysregulation and peer pressure have greater predictive power for the presence of self-injurious behaviors in adolescents compared to the role of digital social networks (H1). Furthermore, these factors are posited to moderate the relationship between childhood trauma and self-injury (H2).

## Methods

The study follows a quantitative approach, with a non-experimental cross-sectional design and correlational-explanatory scope to examine the interaction between variables such as childhood traumatic events, emotional dysregulation, peer pressure, digital social networks, and their related value on self-injurious behaviors and suicidal ideation. The protocol was reviewed and approved by the Ethics Committee in Psychology Research (Ref. No. CEIP-0623-03).

### Participants

The sample was a non-probabilistic, intentional selection of adolescents attending secondary school in Nuevo Leon, Mexico, regardless of gender, and of Mexican nationality. Initially, 402 surveys were collected, and after eliminating incomplete responses or those from individuals not originally from Mexico, the final sample consisted of 351 adolescents: 228 (65%) female and 121 (35%) males. The psychological assessment battery was administered in three public and two private secondary schools. The sample included 113 first-year students (32%), 119 second-year students (34%), and 119 third-year students (34%). The participants' ages ranged from 11 to 15 years, with 8% aged 11, 28% aged 12, 32% aged 13, 30% aged 14, and 1% aged 15 (*M* = 12.88, *SD* = 0.971).

### Procedure

Prior authorization was obtained from the educational institutions, including informed consent from school administrators and parents and informed assent from the participants. These documents outlined the research objectives, confidentiality protocols, voluntary participation, potential risks, and provided contact information for four psychological care institutions and a 24/7 emotional S.O.S. service. Finally, to provide emotional support after addressing the sensitive topic, all participants were presented with an interactive fable designed by the researchers focused on self-injury prevention. The fable offered emotional regulation tools and coping strategies. The fable emphasizes the importance of listening to and understanding emotions, as well as seeking help through friends, acquaintances, or mental health professionals when needed (findings accessible in another article).

### Measurement instruments

#### Inventory of Statements About Self-Injury (ISAS)

ISAS is a self-report measure designed by Klonsky and Glenn (2009) to assess the functions of non-suicidal self-injury (NSSI). Later, Castro Silva et al. (2016) validated it with Mexican college students attending an important public university. The study included 281 women (64.1%) and 152 men (35.1%), with ages ranging from 17 to 34 years (Mage = 20.3). They were studying 27 different programs including Psychology, Engineering, Medicine, Sociology, among others. Regarding the frequency of NSSI, 36.9% had performed the behavior experimentally one to four times in their lives, while 63.1% performed it recurrently, five or more times in their lives. The first section of the scale evaluates the occurrence and frequency of the 12 most common types of NSSI: cutting, severe scratching, biting, hitting oneself forcefully, burning, making shallow skin cuts, interfering with wound healing, rubbing skin against a rough surface, pinching, poking with needles, pulling out hair, and ingesting harmful substances. It also includes an “Other” option to report additional methods not listed. For participants who reported NSSI, five items assessed contextual factors such as the age of onset, the experience of pain during self-injury, whether the behavior was performed alone or with others, the time between the urge to self-harm and the act, and the intention to cease the behavior.

The second section consisted of 39 items evaluating the relevance of 13 potential functions of NSSI rated on a scale from “0-not relevant,” “1-somewhat relevant,” to “2-very relevant”: affect regulation, anti-dissociation, anti-suicide, autonomy, interpersonal boundaries, interpersonal influence, distress marking, peer bonding, self-care, self-punishment, revenge, sensation seeking, and persistence. These functions are grouped into two factors: interpersonal and intrapersonal (Klonsky and Glenn, 2009). In this study, reliability was good (α = 0.82).

#### Paykel Suicide Scale (PSS)

Developed by Paykel et al. (1974) and validated for Latin American adolescents (Baños-Chaparro and Ramos-Vera, 2020), this scale assesses suicidal ideation over the past year. It includes five dichotomous response items (yes/no). The first two items measure thoughts of death, the third and fourth items assess suicidal ideation, and the fifth item addresses suicide attempts. Reliability was satisfactory (α = 0.86) in this study.

#### Peers and Parents Influence Scale (PPI)

Developed by Werner-Wilson and Arbel (2000) and adapted to Mexican Spanish by Magallanes-Lozano et al. (2021). This scale compares the influence of parents vs. peers through 17 items, such as “Overall, I am more influenced by my friends than by my parents,” answered on a Likert scale ranging from “1-strongly disagree” to “7-strongly agree.” Higher scores indicate greater peer influence. Reliability was acceptable (α = 0.68).

#### Emotion Dysregulation Scale-Short (EDS-S)

Adapted by Powers et al. (2015) and validated in Mexico by Contreras Valdez (2017), this self-report instrument consists of 12 items assessing three dimensions of emotional dysregulation: emotional experience (“My emotions overwhelm or burden me”), cognitive (“When I'm upset, everything feels like a disaster or crisis”), and behavioral (“When my emotions are strong, I often make poor decisions”). Responses are on a five-point Likert scale ranging from “0-not at all” to “4-a lot.” The study achieved excellent reliability (α = 0.92).

#### Early Trauma Inventory Self-Report—Short Form (ETI-SR-SF)

Adapted by (Bremner et al. 2007), this self-report instrument includes 27 dichotomous response items (yes/no) that evaluate traumatic experiences before age 18 across five domains: 11 items on general trauma (α = 0.7) “Have you ever experienced a serious injury or illness?”; five items on physical punishment (α = 0.75) “Have you ever been punched or kicked?”; five items on emotional abuse (α = 0.86) “Were you frequently made to feel inferior or ridiculed?”; and six items on sexual events (α = 0.87) “Have you ever experienced someone rubbing their genitals against you against your will?” The total scale reliability was good (α = 0.86).

#### Screening of self-injurious behaviors and suicide related with internet use

Based on a questionnaire used in a study involving parents and children in the United Kingdom to assess the influence of exposure to information about suicide and self-injurious behavior on the internet (Mars et al., 2015), a survey was designed and initially validated through expert content review by a panel of five researchers. This was followed by a pilot study conducted with prior parental consent and adolescent assent. Despite receiving information about the research, 15 parents declined participation, citing the sensitivity of the topic.

A total of 27 adolescents aged 11–16 from the general population in Nuevo Leon, Mexico completed the instrument and participated in cognitive interviews to evaluate the clarity and relevance of the items. Twenty adolescents (74.07%) suggested no changes. The remaining participants proposed specifying internet search engines to avoid confusion and replacing the term “site” with “page.” The final instrument comprises two parts. The first section includes 12 questions about internet use, probing the presence of information on self-injury and suicide as well as interactions with it. Example questions include: (i) “There are digital spaces where suicide is discussed. Have you ever interacted in any of these?” (iii) “Have you ever searched for information on self-injury using an internet search engine (Google, Bing, ChatGPT, etc.)?” (v) “Have you ever used the internet to talk about self-injury with others on a social network?” (x) “Have you ever used the internet to search for content about suicide in videos and/or podcasts?”

Participants who answered affirmatively in the first section were asked to complete the second part of the questionnaire, consisting of two checklists—one for self-injurious behaviors and another for suicidal ideation. They were asked to indicate the types of pages where they usually find such information, choosing from six options: (1) news about individuals who have harmed themselves or died by suicide, (2) social media accounts of individuals who have self-harmed or discuss suicide, (3) general informational pages about suicide and self-injury, (4) pages dedicated to individuals who practice self-injury or have suicidal ideations, (5) support sites offering advice and help, (6) pages providing information on how to self-injury or die by suicide. The answers to these questions could reflect the possible desensitization of adolescents on these issues. The reliability of this instrument was good (α = 0.86).

#### Assessment battery

A survey was prepared using the six measurement scales for virtual administration via Qualtrics. The survey began with informed assent from participants, followed by randomly ordered instruments to counterbalance and avoid fatigue bias. The survey was divided into sections, and participants who did not report traumatic events, self-injurious behaviors, or suicidal ideation were excluded from subsequent sections containing sensitive information.

The total number of items could be reduced to 74 (56.5%) out of the original 131, depending on participant responses. Demographic questions about age, sex, gender, grade level, and nationality were included at the end.

### Statistical data analysis

The statistical software JASP was used to conduct reliability analyses of the instruments and the necessary association analyses to perform multiple linear regression. Self-reports related to self-injurious behaviors, suicidal ideation, and childhood traumatic events report presence and frequency, meaning they are metric or dimensional scales. Therefore, for statistical analyses, they were considered ratio or continuous variables. For the same reason, the correlation was carried out using Spearman's rank coefficient.

A significance level of 95% was applied to determine the predictive value of childhood traumatic events, emotional dysregulation, peer pressure, and digital social networks on self-injurious behaviors and/or suicidal ideation.

## Results

### Prevalence of self-injurious behaviors

The results revealed that 67.52% of the sample, or 237 individuals, reported engaging in non-suicidal self-injury (NSSI) at least once. The average frequency of self-injury per individual was 182.6 instances (*SD* = 488.4; *Mdn* = 23), ranging from 1 to 4,702 occurrences. Of these, 162 (67.7%) were female, and 75 (31.3%) were male (*t* = −3.486, *p* < 0.001, *d* = −0.392).

The following patterns of self-injurious behaviors were observed: 108 participants reported cutting themselves; 177 scratched themselves forcefully; 135 bit themselves; 122 hit themselves forcefully; 50 burned themselves; 146 interfered with the healing of wounds; 112 rubbed their skin; 80 rubbed their skin against rough surfaces; 132 pinched themselves; 76 pricked themselves with needles; 118 pulled their hair; and 29 ingested harmful substances.

Additionally, 23 participants selected the “other” category, reporting behaviors such as: starving themselves, cutting their hair, staying up very late, cutting themselves with a pencil sharpener, injuring their hands, hitting themselves with metal or other objects, breaking objects, crying, hanging themselves, attempting suicide, peeling the skin off their lips, standing under the shower and crying, talking to themselves and hitting their head. All cases where participants reported non-self-injurious behaviors in the “other” category were excluded from the counts but indicated a quantity in their responses.

These data provide a detailed view of the various modalities of self-injurious behaviors present in the sample, with scratching forcefully and interfering with wound healing being the most common forms. Additionally, Table 1 shows the functions attributed by individuals to their self-injurious behaviors.

**Table 1:** Functions attributed to self-injurious behaviors by participants.

**Function**	**Frequency**	**Percentage (%)**
Emotional regulation	22	6.25
Self-punishment	17	4.83
Perseverance	12	3.40
Generation of feelings	11	3.13
Anti-suicide	9	2.56
Autonomy	8	2.28
Self-care	4	1.13
Interpersonal boundaries	3	0.85
Revenge	3	0.85
Peer connection	2	0.57
Distancing from others	2	0.57
Sensation seeking	1	0.28
Interpersonal influence	0	0.00

The table highlights the potential functions of Non-Suicidal Self-Injury (NSSI) identified by the 180 participants who rated their self-injurious experience as “very relevant,” with emotional regulation being the most frequently cited reason. Additionally, 150 participants (83.33%) expressed a desire to stop engaging in these behaviors.

### Prevalence of thoughts of death, suicidal ideation, and suicide attempts

A total of 195 participants (55.56%) reported having *thoughts of death* (i.e., “I wish I were dead”), 162 participants (46.15%) reported *suicidal ideation* (i.e., “I have made plans to take my own life”), and 75 participants (21.3%) reported at least one *suicide attempt* at some point in their lives. Among the 195 participants who reported thoughts of death, 157 (80.05%) also engaged in self-injurious behaviors. Of the 162 participants with suicidal ideation, 141 (87.03%) engaged in at least one self-injurious behavior. Finally, of the 75 participants who reported a suicide attempt, 72 (96%) also reported self-injurious behaviors.

### Prevalence of childhood traumatic events

Regarding experiences of traumatic events at some point in life, 295 participants (84%) had experienced general trauma (Table 2); 272 participants (77.71%) reported having experienced physical trauma in its various forms (Table 3); 250 participants (71.42%) reported having experienced at least one emotional trauma (Table 4) and, finally, 87 participants (24.85%) reported having experienced some form of sexual trauma (Table 5).

**Table 2:** Prevalence of general traumatic events.

**General traumatic events**	**Frequency**	**Percentage (%)**
Exposure to a natural disaster where their life was in danger	59	16.8
Serious accident	70	19.9
Severe injury or illness	47	13.4
Death or severe illness of a parent or primary caregiver	41	11.7
Divorce or separation of parents	39	11.1
Severe injury or illness of a sibling	24	6.8
Death or severe injury of a friend	6	1.7
Witnessing violent acts against others	6	1.7
Family member with a mental or psychiatric illness	2	0.6
Parents or primary caregiver with alcoholism or drug abuse	1	0.3
Witnessing a murder	1	0.3

**Table 3:** Prevalence of physical violence traumatic events.

**Physical violence traumatic events**	**Frequency**	**Percentage (%)**
Slapped with an open hand	61	17.4
Burns of some kind	60	17.1
Punches or kicks	71	20.2
Hit by a thrown object	55	15.7
Shoves	25	7.1

**Table 4:** Prevalence of emotional traumatic events.

**Emotional traumatic events**	**Frequency**	**Percentage (%)**
Feeling ridiculed	54	15.4
Feeling ignored	52	14.8
Hearing that they are not good people	40	11.4
Being treated coldly, carelessly, or made to feel unloved	50	14.2
Considering that their parents have failed to meet their needs	54	15.4

**Table 5:** Prevalence of sexual traumatic events.

**Sexual traumatic events**	**Frequency**	**Percentage (%)**
Being intentionally touched in their private parts in an uncomfortable way.	32	9.1
Someone rubbing their genitals against them against their will.	23	6.6
Being forced to touch another person's private parts.	14	4.0
Being subjected to sexual violence via genital.	9	2.6
Being subjected to sexual violence via oral.	6	1.7
Being forced to kiss someone in a sexually rather than affectionally.	3	0.9

### Social media

It was found that at least 127 (36.1%) individuals have interacted online with content related to non-suicidal self-injury or suicide. Similarly, the results from the questionnaire on exposure to self-injurious behaviors on social media revealed the frequency of pages visited by young people, either related to self-injurious behaviors or suicide (Table 6), with news about people who self-harm or have taken their own lives being the main form of exposure in both cases.

**Table 6:** Ways of viewing self-injury and suicidal content on social media.

**Content of NSSI on social media**	**Self-injury**		**Suicide**	
	**Frequency**	**Percentage (%)**	**Frequency**	**Percentage (%)**
News about people who have harmed themselves	90	25.56	98	27.92
Social media accounts of people who have harmed themselves	64	18.2	62	17.67
Interacted with pages containing general information about self-injury	64	18.2	30	8.54
Pages dedicated to people who engage in self-injury	39	11.11	41	11.68
Pages and/or support groups offering advice and help for people with self-injury	40	11.39	51	14.53
Pages with information on how to self-harm	41	11.68	39	11.11

### Inferential analysis

#### Correlation analysis

In Table 7, the results of the Spearman correlation analysis conducted to examine the associations between the studied variables are shown.

**Table 7:** Spearman correlations between Self-Injurious Behaviors with the rest of studied variables.

**Variable**	**rho**	***p***
Suicide ideation	0.498	< 0.001
Suicide attempts	0.404	< 0.001
Peer influence	0.257	< 0.001
Emotional dysregulation	0.435	< 0.001
General traumatic events	0.389	< 0.001
Physical violence traumatic events	0.322	< 0.001
Emotional traumatic events	0.465	< 0.001
Sexual abuse traumatic events	0.323	< 0.001
Interaction in digital social networks with self-injury	0.410	< 0.001
Interaction in digital social networks with suicide	0.405	< 0.001

According to these results, the variables correlate positively with self-injurious behaviors.

#### Multiple linear regression analysis

Subsequently, a multiple linear regression analysis was conducted with the variables of suicide ideation, suicide attempts, emotional dysregulation, peer influence, exposure to self-harm and suicide content on social media, and childhood traumatic events to verify the predictive value of the presence of self-injurious behaviors (Table 8).

**Table 8:** Multiple linear regression analysis for predictors of self-injurious behaviors.

**Variable**	**β**	***t***	***p***	**95% CI**
Suicide ideation	0.14	1.44	0.066	[−5.48; 166.60]
Suicide attempts	−0.03	−0.48	0.635	[−186.36; 113.87]
Parental influence	0.06	1.12	0.265	[−20.08; 72.78]
Peer influence	0.05	0.93	0.351	[−27.55; 77.28]
Emotional dysregulation	−0.02	−0.38	0.701	[−61.72; 41.58]
General traumatic events	0.11	1.56	0.121	[−34.76; 297.86]
Physical violence traumatic events	−0.02	−0.25	0.802	[−119.52; 92.42]
Emotional traumatic events	0.07	0.92	0.358	[−60.90; 168.17]
Sexual traumatic events	−0.08	−1.48	0.140	[−451.47; 63.94]
Interaction in digital social networks with self-injury	0.15	2.03	0.043	[2.11; 128.08]
Interaction in digital social networks with suicide	0.26	3.29	0.001	[43.67; 173.92]

*F*_(10, 335)_ = 10.31, *R*^2^ = 0.25.

None of the variables predict self-harming behavior, except for the two related to interaction with content in digital social networks self-injury (*p* = 0.043) and suicide (*p* = 0.001) content in digital social networks; suicide ideation is marginally significant (*p* = 0.066).

### Hypothesis testing

The H1 hypothesis, which stated that emotional dysregulation and peer pressure would predict self-injurious behaviors more significantly than digital social media exposure, was rejected. Exposure to content about self-injury and suicide on social media emerged as the two most predictive variables for the presence of self-injurious behaviors. Additionally, H2 could not be confirmed, as emotional dysregulation and peer pressure did not predict self-injurious behaviors in this sample.

## Discussion

In this research, four major findings were identified: the high prevalence of self-injurious behaviors, the role of exposure to suicide-related content on social media, the desensitization of this topic, and the importance of addressing this issue with adolescents from an empathetic and human perspective.

The statistics regarding the incidence of self-injurious behaviors in adolescents (67.52%) represent double the previously reported figures (13–45%) in global and national studies (Campo Arias, 2022; Plener et al., 2016; Vega et al., 2018); highly prevalent in women (Farkas et al., 2024; Wang et al., 2022; Xiao et al., 2022) who self-injure twice (67.7%) as much as men (31.3%). Furthermore, more than 50% of the participants reported having thoughts of death and 46% informed about suicidal ideation, most of which were accompanied by non-suicidal self-injury (NSSI). This pattern is reflected in previous research (Gillies et al., 2018; Ospina Gutiérrez et al., 2019), which highlights the strong relationship between these two variables. Particularly, when self-injury is severe or repeated over an extended period, there is a higher risk of experiencing suicidal ideation or attempts.

The second major finding reveals that, although previous research has documented the relationship between self-injurious behaviors and childhood traumatic events (Persaud et al., 2023; Quiroga-Garza and Almela-Ojeda, 2022; Romero-Acosta et al., 2018), emotional dysregulation (Bautista Hernández et al., 2022; Gratz and Roemer, 2004; Klonsky, 2007; David Klonsky and May, 2015; Peh et al., 2017; Wu et al., 2021), and peer pressure (Copeland et al., 2021; Smith-Gowling et al., 2018; Wu et al., 2019, 2021; You et al., 2016), the results of this study introduce an additional variable. Adolescents' exposure to self-injury and suicide content on digital social media showed predictive power regarding the presence of these behaviors.

The results emphasize the related value of digital social media and the constant exposure to content related to self-injury and suicide. There were cases where adolescents (18) mentioned having engaged in some self-injurious behavior, but in the “other” section, they did not consider it as self-injury. It would be important to analyze in future studies why this distinction was made, as it could be related to desensitization about the topic. This desensitization might lead to a misperception of the severity of self-injurious behaviors and suicidal ideation or stem from a lack of knowledge on the subject (Biernesser et al., 2020; Dekel et al., 2024; López Martínez, 2020; Marchant et al., 2017; Moss et al., 2023). These potential reasons could make it more difficult for adolescents and young people to recognize the importance of seeking help or intervening when necessary.

The findings of the present study contribute to the evidence of research conducted on self-injurious behaviors in adolescence. In our study, conducted shortly after the confinement and subsequent social distancing due to the SARS-CoV-2 pandemic, we found a concerning nearly 2 fold increase in NSSI, with over 50% of participants also reporting thoughts of death and suicidal ideation. The reasons commonly reported by adolescents in other studies—traumatic childhood events (Romero-Acosta et al., 2021; US Department of Veterans Affair, 2022), emotional dysregulation (Bautista Hernández et al., 2022; Frías et al., 2012; Gratz et al., 2018; Hervás, 2011; Hervás and Moral, 2017; Hervás and Vázquez, 2006; Klonsky, 2009; Peh et al., 2017; Wu et al., 2021), and peer pressure (Copeland et al., 2019; Hasking et al., 2013; Holly, 2011; Jarvi et al., 2013; Prinstein et al., 2010; Walsh and Muehlenkamp, 2013; Smith-Gowling et al., 2018; You et al., 2016; Zaragozano, 2017)—joined to exposure to self-injury and suicide content on digital social networks (Lavis and Winter, 2020; Sampasa-Kanyinga and Lewis, 2015; Sedgwick et al., 2019; Twenge and Campbell, 2019). The predictive power of these behaviors was the highest for both suicide (*t* = 3.286, *p* = 0.001) and self-injury (*t* = 2.033, *p* = 0.043). Cutting, forceful scratching, and interfering with wound healing—the most common forms of NSSI (Abdou et al., 2024; Klonsky, 2011; Pérez et al., 2021)—seem to indicate a pattern: after injuring themselves, they keep the wound open as a form of self-regulation or self-punishment, the two most frequently reported motives. If we add to this that the experience of both physical and psychological pain is associated with sensory perception and emotional response (Gilam et al., 2020; Gross and Barrett, 2011; Shackman and Wager, 2019), emotional desensitization can emerge from repeated exposure to self-harm content on digital social networks (Biernesser et al., 2020; Dekel et al., 2024; López Martínez, 2020; Marchant et al., 2017; Moss et al., 2023). Connecting digitally with other adolescents who resort to self-inflicted behaviors to reduce pain—the main way of exposure on networks was to news about people who self-injury or have suicided—makes it an increasingly less discordant and unpleasant mental, emotional, and psychophysiological topic (Miles-Novelo and Anderson, 2020).

Interacting with peers who openly engage in and share self-inflicted behaviors for self-verification and a sense of belonging indicates the path forward. If the adolescent is in a permanent state of feeling unable to self-regulate, feeling alone in front of a screen that keeps them in a limited state of mobilization, it is necessary to work on reconnecting them to active in- person social life where they may interact with peers and significant adults—parents and teachers. Being in tune with others allows for co-regulation and provides a sense of wellbeing, security, and belonging.

### Limitations of the study

Limitations were presented in this research. The first occurred when requesting authorization from schools: five institutions did not respond, and three others withdrew from the process, citing that their students were not prepared to receive information on the topic or that the profiles of the individuals we sought were not present in their institutions. Nonetheless, these school participation challenges and participant response inconsistencies, would help improve the generalizability of the findings, the data analysis underscores the urgency of addressing self-injurious behaviors and suicidal ideation in adolescents.

Another limitation arose in the data analysis. Contradictions were found in the participants' responses, where they either failed to recognize their self-injurious behaviors or left the survey incomplete. Some participants indicated they did not want to answer certain questions.

Furthermore, there was a gender imbalance in the people who answered the scales, as most responses were obtained from women, with 65% compared to 35% men. Another limitation lies in the lack of inquiry into the socioeconomic status of the participants, as students from three public and two private high schools participated. Due to the foregoing, the results cannot be generalized.

In addition, qualitative research is crucial to analyze the intention behind each self-injury according to the participants' own definitions: What makes habits or emotional responses to self-injury a way to get relief? Which self-injurious behaviors have been insufficiently significant to classify as such? Some adolescents noted they did engage in self-injury, providing examples like crying, staying up late, and not eating, as these behaviors had a destructive intent for them, which should be considered when addressing the issue.

### Final considerations

Based on the findings of this study, an integrated approach to self-injurious behaviors and suicide is emphasized, involving close collaboration between parents, educators, professionals, and society at large to prevent them and create a safe environment for adolescents, particularly girls.

It is important to note that research on the desensitization of this issue is scarce, even though it may constitute a significant factor in normalizing these behaviors. A deeper focus on this topic is recommended, with prevention efforts aiming to raise awareness. Tools such as psychoeducation should be implemented, informing adolescents about the risks and warning signs of self-injury and suicide, as well as promoting open communication and emotional support in school environments. Furthermore, regulating access to harmful online content and promoting responsible social media use should be prioritized.

The high reported prevalence of self-injurious behaviors is accompanied by the distress and desire of adolescents to have the power to stop resorting to self-destructive behaviors. This would require providing emotional support strategies grounded in compassion and sensitivity. These resources can be implemented in various institutions to help adolescents create sustainable and healthy coping mechanisms through collaborative efforts. Parents, teachers, administrators, health professionals, organizations, and society at large must join forces to eliminate prejudices and stigmas about this issue, staying vigilant to identify warning signs and foster positive change that counters the current situation. Without a doubt, this is a crucial public agenda issue for the wellbeing of the adolescent population.

## Conclusions

Understanding the predictive relationship between various personal and social variables and self-injurious behaviors in adolescence presents a significant challenge. Future research should explore the factors contributing to the desensitization toward self-injurious behaviors and suicidal ideation among adolescents. Specifically, studies could investigate the link between this desensitization, a misperception of severity, and a lack of knowledge on the subject.

Understanding these variables is crucial, as they may make it more difficult for young people to recognize the importance of seeking help or intervening when necessary. This line of inquiry could inform the development of targeted educational programs and interventions aimed at improving mental health literacy and promoting help-seeking behaviors.

Future research should also conduct a deeper qualitative analysis of adolescent self-harm, exploring the subjective intention behind each behavior from a participant-centered perspective. It is crucial to understand why certain maladaptive habits are considered self-harm by some adolescents but not by others. This research will offer valuable insight into the cognitive frameworks adolescents use to define and experience self-harm, which is essential for creating more effective, empathetic and compassion-based interventions.

Finally, a collaborative effort from all sectors—parents, educators, and healthcare professionals—is essential to eliminate the prejudice and stigma surrounding this issue. By uniting to identify warning signs, we can create a positive and lasting change to better ensure the wellbeing of the adolescent population.

## Data Availability

The raw data supporting the conclusions of this article will be made available by the authors, without undue reservation.
